# Clinical and Functional Characterization of a Novel Mutation in *AVPR2* Causing Nephrogenic Diabetes Insipidus in a Four-Generation Chinese Family

**DOI:** 10.3389/fped.2021.790194

**Published:** 2021-12-09

**Authors:** Shusen Guo, Shimin Wu, Zhuxi Li, Lianjing Huang, Di Zhan, Cai Zhang, Xiaoping Luo

**Affiliations:** Department of Pediatrics, Tongji Hospital, Tongji Medical College, Huazhong University of Science and Technology, Wuhan, China

**Keywords:** *AVPR2*, congenital nephrogenic diabetes insipidus, novel mutation, functional analysis, short stature, growth hormone treatment

## Abstract

**Background:** Congenital nephrogenic diabetes insipidus (CNDI) is a rare inherited disease that is caused by mutations in *arginine vasopressin receptor 2* (AVPR2) or *aquaporin 2* (*AQP2*). Functional analysis of the mutated receptor is necessary to verify the impact of the mutation on receptor function and suggest some possible therapeutic strategies for specific functional defects.

**Methods:** Family history and clinical information were collected. Whole-exome sequencing and sanger sequencing were performed to determine the potential genetic cause of diabetes insipidus. The identified variant was classified according to the American College of Medical Genetics (ACMG) criteria. Bioinformatic analysis was performed to predict the function of the identified variation. Moreover, wild-type and mutated AVPR2 vectors were constructed and transfection to HEK-293T cells. Immunofluorescence experiments were performed to investigate the expression and localization of the mutated protein and cAMP parameter assays were used to measure its activity in response to AVP.

**Results:** The heights of the adult members affected with polyuria and polydipsia were normal, but all affected children had growth retardation. Next-generation sequencing identified a novel mutation in *AVPR2* gene (c.530T > A) in this family. Bioinformatic analysis indicated that the mutation in AVPR2 changed the hydropathic characteristic of the protein and was probably deleterious. Although immunofluorescence showed that the mutated AVPR2 was normally expressed in the cell surface, the intracellular cAMP concentration stimulated by AVP was significantly lower in cells transfected with mutated AVPR2 than cells transfected with wild-type AVPR2. Based on the ACMG criteria, the novel c.530T > A variant of the *AVPR2* gene was likely pathogenic and the affected family members were diagnosed as CNDI. After the confirmation of the diagnosis, the proband was treated with compound amiloride hydrochloride and rhGH, the symptoms of polyuria, polydipsia and growth retardation were all improved.

**Conclusion:** These findings suggested that the novel mutation in *AVPR2* (c.530T > A) was a true disease-causing variant with mild effects, which could be classified as a type III mutant receptor. Moreover, investigations of the function of growth hormone axis could be important for the pediatric CNDI patients with extreme short stature, and rhGH treatment might improve the final adult heights in these patients.

## Introduction

Congenital nephrogenic diabetes insipidus (CNDI) is a relatively rare inherited disease with an estimated incidence of ~1 in 1,00,000. It's characterized by the failure of the kidneys to concentrate urine due to renal resistance to antidiuretic hormone (AVP) (1, 2). The cardinal clinical symptoms are polyuria with hyposthenuria and polydipsia. However, in infants, faltering growth, frequent vomiting and hyperthermia represent the more common symptoms (3–5). The current management strategies for CNDI, including treatment for dehydration and hypernatremia, thiazide diuretics, prostaglandin synthesis inhibitors, and a low-salt diet, are sub-optimal at best; patients still experience significant polyuria and polydipsia and require monitoring for the development of hydroureter (6). Thus, further studies on the molecular mechanism and function of variation proteins in CNDI are needed that may reveal potential new treatment strategies (7). Approximately 90% of CNDI cases are X-linked recessively inherited and are caused by mutations in the *arginine vasopressin receptor 2* (*AVPR2*) gene. The remaining 10% cases are caused by variations in *water channel protein aquaporin 2* (*AQP2*) gene, which are inherited in an autosomal dominant or recessive manner (8).

*AVPR2* gene is located in chromosome Xq28 and consists of three exons. It encodes the AVPR2 protein (9) which has a complex three-dimensional structure and shares the typical structure of G protein-coupled receptors (GPCRs). AVPR2 is mainly expressed in the principal cells of the renal collecting tubules (7). It couples to the pituitary hormone arginine vasopressin (AVP), thus promoting the cAMP/PKA-mediated trafficking of AQP-2 into the luminal membrane. AQP-2 serves as a water channel protein that play a major role in allowing water to freely enter via the medullary osmotic gradient. Therefore, AVPR2 is essential for the kidney's ability to concentrate the urine and maintain water balance (9–11).

Numerous inactivating or activating variations of *AVPR2* have been identified and are closely associated with human diseases (1, 7). To date, approximately 290 *AVPR2* gene variations supposed to cause CNDI have been reported in the Human Gene Mutation Database (HGMD Professional 2021), most of which are missense variations. The detection of a missense variation in *AVPR2* gives a hint for a possible pathogenic cause of CNDI. Additionally, functional analysis is necessary to verify the impact of the mutation on receptor function and may suggest possible therapeutic strategies for specific functional defects (12–17). Therefore, it is important to understand the effects caused by a given variation in detail. Here, we report the clinical characteristics and genetic variation in *AVPR2* gene that is associated with CNDI in a four-generation Chinese family. Moreover, the pathogenic consequences of the identified mutation were analyzed by using bioinformatic approaches and *in vitro* cellular experiments.

## Materials and Methods

### Family Enrollment and Ethics Statement

This study enrolled a four-generation Chinese family from China. The proband was a 3-year-old male patient who was suspected to be afflicted with NDI and growth retardation, from pediatric clinic of Tongji Hospital. The medical history and clinical manifestations of family members were collected. The human study was approved by the Ethics Committee of Tongji Hospital, Tongji Medical College, Huazhong University of Science and Technology (TJ-IRB20201016). The study was performed according to the declaration of Helsinki and written informed consent was obtained from each patient; for patients below 18 year of age the written informed consents were obtained by the patient's parents.

### Genomic DNA Sequencing and Bioinformatic Analysis

Peripheral blood was collected from the proband and his family members and genomic DNAs were isolated using QIAamp DNA Blood Mini Kit (Qiagen, Germantown, MD). Whole exome sequencing (WES) was performed in the proband and his parents concurrently by MyGenostics (Beijing, China) using the Illumina HiSeq X ten system. Genetic variants and their segregation in the family were confirmed by Sanger sequencing. Briefly, Genomic DNAs was fragmented to 100–700 bp and then fragments measuring of 150–200 bp were selected with magnetic beads. Whole-exome capture was carried out using the GenCap WES capture kit (MyGenostics, Beijing, China) followed by massively parallel sequencing by Illumina HiSeq X ten sequencer. High-quality reads were mapped to the human reference genome hg19 using BWA (http://bio-bwa.sourceforge.net). Variants, including SNPs, indels and block substitutions, were analyzed by GATK (https://software.broadinstitute.org/gatk), and annotated using the 1000 Genomes Project (http://www.1000genomes.org), bSNP (http://www.ncbi.nlm.nih.gov/projects/SNP), HGMD (http://www.hgmd.cf.ac.uk) and the MyGenostics local database. Sanger sequencing was performed was performed to confirm the potentially pathogenic variants.

The online tools SIFT and PROVEN (http://provean.jcvi.org/index.php), PolyPhen-2 (http://genetics.bwh.harvard.edu/pph2), Mutation Taster (http://www.mutationtaster.org), were used to predict the pathogenicity of identified variants. Hydropathic characters for amino acid changes were displayed with Kyte-Doolittle Hydropathy Plot online software (http://gcat.davidson.edu/DGPB/kd/kyte-doolittle.htm).

### Plasmid Construction

The Flag-tagged wild-type *AVPR2* (NM_000054) and mutation *AVPR2* (c.530T > A) plasmids were constructed by subcloning each target gene's full-length cDNA into lentiviral vector (PCDH-CMV-MCS-EF1-copGFP-T2A-Puro) which was verified by the dual-enzyme digestion and PCR amplification by Vigene Biosciences (Shandong, China). Wild-type and mutation *AVPR2* were verified by DNA sequencing.

### Cell Culture and Transfection

Human embryonic kidney 293T (HEK-293T) cells were cultured in DMEM supplemented with 10% fetal bovine serum and penicillin-streptomycin (1:100, Boster, China) at 37°C in a humidified 5% CO_2_ incubator. HEK-293T cells were transfected with wild-type or mutant AVPR2 plasmids or empty vector using Lipo3000 Reagent (Invitrogen, USA) according to the manufacturer's instructions.

### Immunofluorescence

HEK-293T cells were seeded on cover slips and transfected with wild-type and mutated vector. Forty eight hours after transfection, cells were fixed with 4% paraformaldehyde (PFA), washed with PBS and permeabilized for 15 min with Triton X-100. For immunofluorescence staining, cells were incubated with the primary antibody against Flag-tagged epitope-specific mouse monoclonal antibody (1:100, 8146, CST, USA) and Calnexin (CNX) rabbit monoclonal antibody (1:100, 2679, CST, USA), followed by Donkey anti Rabbit IgG (H&L) Alexa Flour 647 (1:500, ab150075, Abcam, USA) and Donkey anti Mouse (H+L) Alexa Fluor 594 (1:500; A21203, Thermofisher, USA) fluorescent secondary antibody at room temperature for 1 h. Finally, all the cells were stained with 1 μg/mL 4′,6-diamidino-2-phenylindole (DAPI; Sigma-Aldrich) to visualize cell nuclei. Images were taken with a confocal laser-scanning microscope (NIKON Eclipse Ci) and processed using the CaseViewer software (3D HISTECH Ltd., Hungary).

### Intracellular cAMP Measurement and Statistical Analysis

HEK-293T cells were seeded on 24-well plates for 1.5× 10^4^ per well. Forty eight h after transfection, cells were stimulated with various AVP (ab120175, Abcam, USA) concentrations as 10-fold dilution series (1 nM−10 μM) for 1 h at 37°C in 5% CO_2_ incubator. The cellular cAMP content was measured using the cAMP parameter assay kit (KGE002B, R&D systems, USA) according to the manufacturer's instructions. cAMP accumulation data were analyzed using GraphPad Prism version 8.4.3 (GraphPad Software, San Diego, CA). One-way analysis of variance (ANOVA) with Tukey's multiple comparisons test was used for multiple comparisons of normally distributed data. Data were expressed as the means ± standard error of the mean (SEM). Significant difference was defined as *P* < 0.05.

## Results

### Familial Characteristics

The proband, a 3-years old boy, visited pediatric clinic of Tongji Hospital, due to growth retardation and excessive fluid intake and urine output. His daily fluid intake was 6,000 mL and his urine output was 6,000 mL with a nocturia frequency of more than three times per day. His weight was 10 kg (<3rd percentile) and his height was 83 cm (<3rd percentile, −3.89 SDS). No facial or skeletal malformations was observed in the proband.

The boy was born as the first child of non-consanguineous parents with a birth weight of 3,700 g (85th percentile) after an uncomplicated pregnancy at 41 weeks. At the age of 5 months, he was admitted to the local hospital for fever (38°C) and frequent vomiting. He was unresponsive to physical cooling and antipyretic drugs, but his body temperature dropped to normal after drinking more water and perspiration. His urine examination revealed low urine specific gravity (<=1.005). At the age of 11 months, he was diagnosed as short stature with a height of 66 cm (<3rd percentile, −3.58 SDS). His bone age was <6 months and his serum IGF-1 concentration was significantly low (<25, normal 51–303 ng/mL). At the age of 2 years old, he developed polyuria, and polydipsia with a daily fluid intake of 2,000–3,000 mL.

Laboratory examinations showed low urine specific gravity (1.001) and low urine osmolality (71 mOsm/kg), with normal blood sodium and chloride ion levels, suggesting a diagnosis of diabetes insipidus (Table 1). His 24 h urine volume was 5,500 mL and urine sodium, chloride, calcium and inorganic phosphate concentrations were low (Table 1). Ultrasound showed normal sonography of the heart, liver, gallbladder, pancreas, spleen and kidney. His parents refused water deprivation and desmopressin tests. His serum peak growth hormone (GH) concentration was lower than 10 ng/mL at stimulation tests (detail data was lost), and his IGF-1 levels was <25 ng/mL.

**Table 1:** Laboratory examinations.

**Item**	**Result**	**Reference**
Urine specific gravity	<=1.005	1.010–1.025
Urine osmolality, mOsm/kgH_2_O	71	600–1000
Urinary Na, mmol/L	20.1	-
Urinary K, mmol/L	4.42	
Urinary Cl, mmol/L	12.5	-
Urinary Ca, mmol/L	0.26	2.5–8.0
Urinary P, mmol/L	1.4	12.9–43.9
Urinary Mg, mmol/L	0.56	1.67–5.67
Blood Na, mmol/L	138.1	136–145
Blood Cl, mmol/L	99.2	99–110
Blood K, mmol/L	4.34	3.5–5.1
24 h Urinary Na, mmol/24 h	110.55	40–220
24 h Urinary K, mmol/24 h	24.31	25–125
24 h Urinary Cl, mmol/24 h	68.75	110–250
24 h Urinary Ca, mmol/24 h	1.43	2.5–8.0
24 h Urinary P, mmol/24 h	7.7	12.9–42
24 h Urinary Mg, mmol/24 h	3.08	2.5–8.5

**Figure 1:**
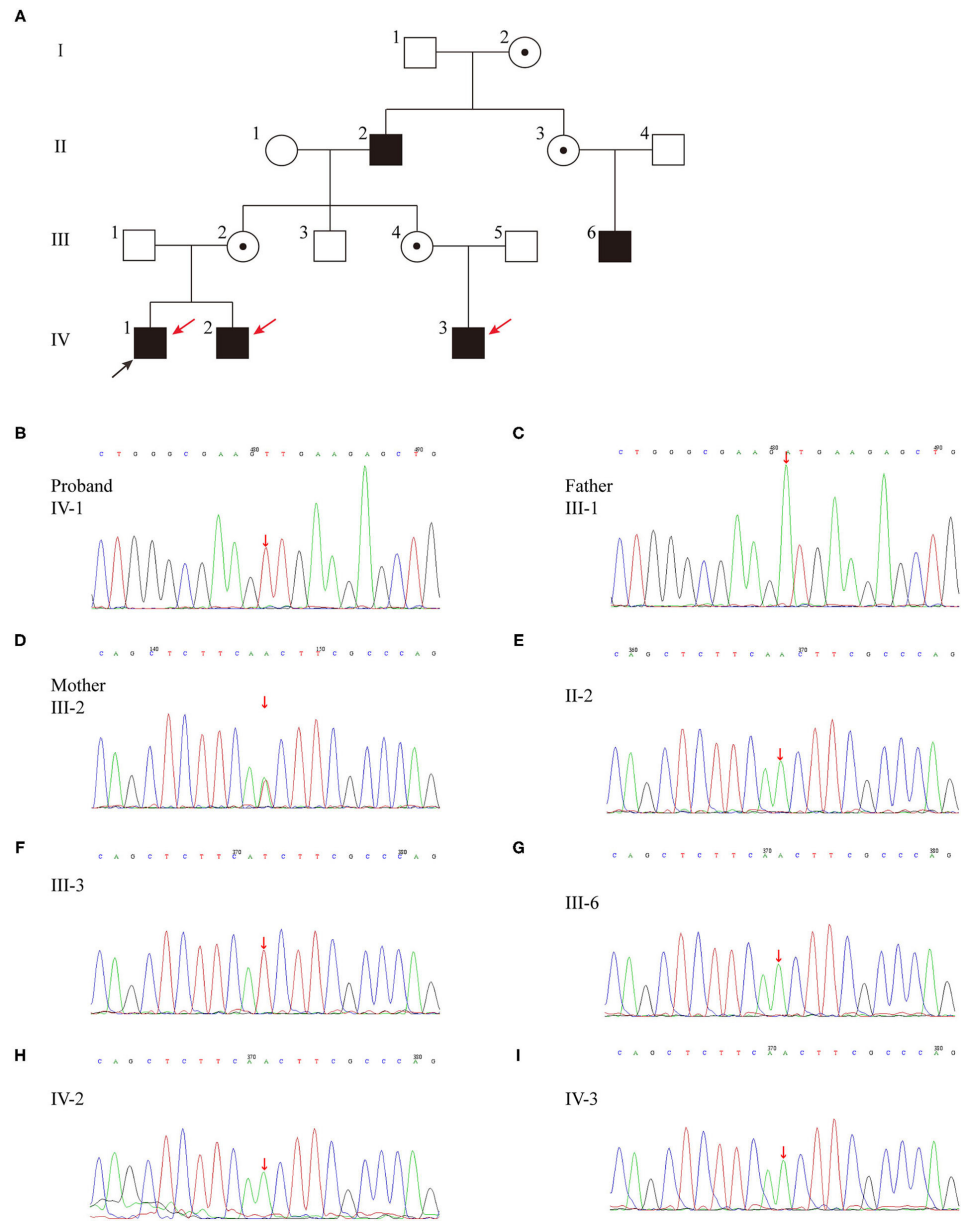
**(A)** Pedigree of the family affected with X-linked recessive inherited NDI. Family members are identified by generations and numbers. Squares indicate male family members; circles, female members; open symbols, unaffected members; black symbols, the affected with polyuria and polydipsia members; circles with a central dot, obligatory carriers; red arrows, the affected with growth retardation members; black arrow, proband. **(B–I)** Sequencing results of the AVPR2 mutation. Sequence chromatograms indicate a T to A transition of nucleotide 530.

The familial pedigree was shown in Figure 1A. The proband's maternal grandfather (case II-2), his mother's paternal cousin (case III-6) and his maternal cousin brother (case IV-3) had a long history of polyuria and polydipsia without any investigation and treatment. The height of case II-2 (174 cm, +0.2 SDS) and case III-6 (178 cm, +0.87 SDS) were normal, while case IV-3 had growth retardation with a height below −2 SDS. The brother of the proband (IV-2) was 11 moth old at visit. He had polydipsia and polyuria, with a fluid intake of 1,500 mL and urine output of 1,400 mL. Moreover, case IV-2 also had growth retardation (69 cm, <3rd percentile, −2.42 SDS) with normal lower limit IGF-1 level (64.81 ng/mL). All of the affected patients were males, in line with the characteristics of X-linked genetic diseases.

### Genetic Analysis

Whole exon genomic analysis of the proband revealed a missense variant in exon2 (c.530T > A) of *AVPR2* gene, which causes a change from isoleucine to asparagine (p.I177N) (Table 2; Figure 1B). This variant appeared to be novel, which is not included in the databased mentioned above, and not reported in normal populations. Molecular analysis disclosed no other variants of clinical significance in AVP or central diabetes insipidus (CDI) or CNDI related genes. There was no variant of *AVPR2* detected in the father of the proband (case III-1) (Figure 1C), while the mother (case III-2) was a heterozygous carrier of the c.530T > A variant (Figure 1D). This variant appeared to be novel, which is not included in the databased mentioned above, and not reported in normal populations. Further analysis showed the same variation was found in affected case II-2, III-6, IV-2 and IV-3 (Figures 1E,G-I). No *AVPR2* variation was detected in phenotypic normal male member (Figure 1F).

**Table 2:** Mutations detected by Next Generation Sequencing.

**Gene**	**Nucleotide variation (exon number)**	**Amino acid change**	**Homo/Het/Hemi (hereditary mode)**	**Associated disease**	**Source of variation**
AVPR2	c.530T > A (exon2)	I177N	Hemi (XR)	CNDI	M
SERAC1	c.1933C > T (exon17)	R645C	Het (AR)	MEGDEL syndrome	F
SERAC1	c.22G > A (exon2)	V8I	Het (AR)	MEGDEL syndrome	M
LRP2	c.92C > T (exon2)	A31V	Het (AR)	Donnai–Barrow syndrome	F
LRP2	c.3836A > C (exon17)	D1279A	Het (AR)	Donnai–Barrow syndrome	M

*hemi, hemizygote; Het, heterozygous; XR, X-linked recessive inheritance; AR, autosomal recessive inheritance; MEGDL syndrome, 3-methylglutaconic aciduria, deafness, encephalopathy, leigh-like syndrome; F, father; M, mother*.

### Bioinformatic Analysis

**Figure 2:**
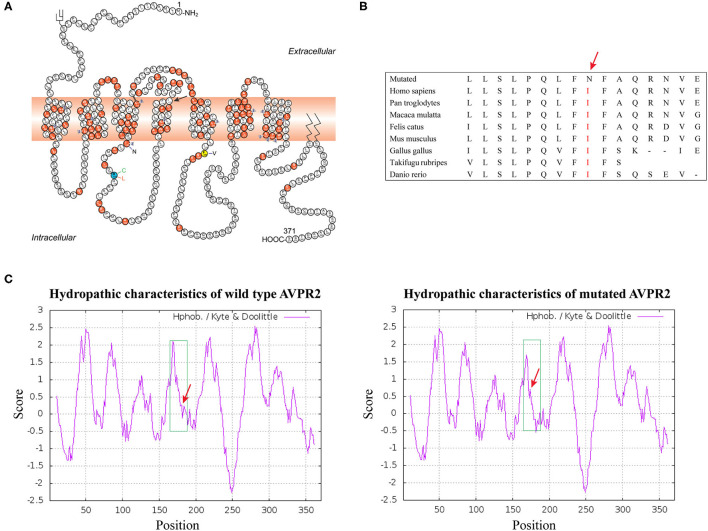
**(A)** Schematic representation of the AVPR2 [The figure is an excerpt from Bichet et al. (4)]. A solid symbol indicates a codon with a missense or nonsense mutation; a number (within a triangle) indicates a different mutation on the cDNA level affecting the same codon; other types of mutations are not indicated on the figure. Mutation analyzed in this study is shown with arrow. **(B)** Analysis of the mutation and protein domains of AVPR2 and alignment of multiple AVPR2 protein sequences across species. The affected amino acid I177 is located in the highly conserved amino acid region in different mammals. Red letters show the I177 site. **(C)** The hydropathic character of the changes in the mutant protein indicated a higher hydrophobicity than that of the WT.

The effect of the missense mutation in p.I177N was predicted to be deleterious by PROVEN with a score of −6.97 (a score ≤-2.5 is considered a deleterious variant) and by SIFT with a score of 0, as well as by PolyPhen-2(probably damaging, score 1.000). This was also predicted to be a disease-causing mutation by Mutation Taster with a score of ~0.9999. Functionally, the c.530T > A transition resulted in a replacement of a hydrophobic amino acid by a hydrophilic amino acid in the hydrophobic transmembrane domain, which changed the hydropathic character of the mutant protein (Figure 2C). Taken together, the bioinformatic analysis revealed that the p.I177N mutation was a novel disease-causing mutation.

### Functional Study/Analysis

#### Localization and Maturation AVPR2

**Figure 3:**
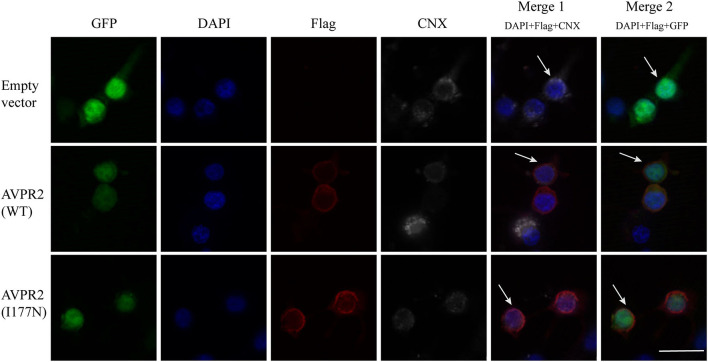
Cellular localization of arginine vasopressin receptor 2 (AVPR2)-I177N. The empty vector or flag tagged AVPR2 or AVPR2-I177N expression vector was transfected into HEK 293T cells. The transfection effect was observed by the expression of green fluorescence protein (GFP), which is not in conjunction with AVPR2. Calnexin (CNX), a marker of ER, marked the ER membrane were shown gray, and the flag tagged AVPR2 proteins were in read. Cell nuclei were counterstained by DAPI (blue). Both single staining and merged images are shown. Both the transfected wild-type and I177N mutant AVPR2 proteins were successfully expressed and could be localized outsider ER, as indicated by the white arrows in the merge 1, and they both could localize on the edge of GFP staining, as showed by the merge 2. The scale bar in the image represents 20 μm.

Gene mutations may affect proper protein folding and result in trapping the receptor in the endoplasmic reticulum (ER). Both the constructed wild-type and mutant AVPR2 were successfully expressed in the 293T kidney cells. To visualize the location where the intracellularly retained receptors accumulated, Flag-tagged wild-type AVPR2 and mutated AVPR2 (p.I177N) were both co-stained with calnexin (CNX), a known marker of ER. Both the transfected wild-type and I177N mutant AVPR2 proteins were successfully expressed and could be localized outside ER, as indicated by the white arrows in the merged images (Figure 3, merge 1). They both could be localize on the edge of GFP staining, which was not in conjunction with AVPR2 and diffusely distributed in cells (Figure 3, merge 2). These results suggested similar localization and maturation of mutated AVPR2 to that of the wild-type protein, indicating that the underlying pathogenesis of the AVPR2 (p.I177N) might not be due to the expression disorder, ER retention, or constitutive endocytosis, but the functional anomaly of the mutated receptor.

#### Evaluation of Response to AVP

**Figure 4:**
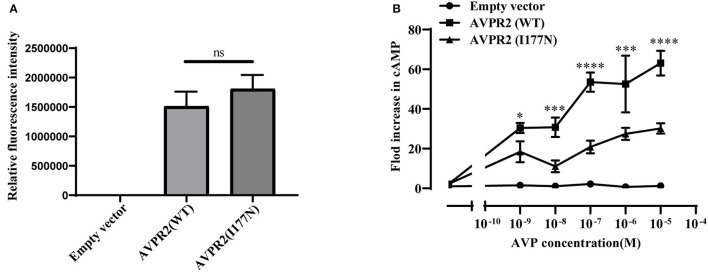
**(A)** Relative fluorescence intensity of Flag-tagged AVPR2. Data were expressed as mean ± SEM, ns means no significant difference. **(B)** AVP-induced cAMP accumulation mediated by the wild-type AVPR2 (AVPR2-WT), mutant AVPR2(AVPR2-I177N) and empty vector. Data were expressed as mean ± SEM, *P < 0.05, ***P < 0.001, ****P < 0.0001.

To investigate the function of the mutated AVPR2, the ability to generate cAMP in response to AVP was evaluated in HEK-293T cells transfected with wild-type or mutated AVPR2 vectors. The expression levels of flag-tagged AVPR2 were comparable different between wild-type and mutated AVPR2 vectors (Figure 4A). HEK-293T cells transfected with empty vectors showed no response of intracellular cAMP levels to the simulation of AVP, suggesting no endogenous AVPR expression in the cells. After transfected with wild-type AVPR2 vectors, HEK-293T cells responded dramatically to AVP, indicated by a significantly increase of intracellular cAMP levels. However, HEK-293T cells transfected with mutated AVPR2 vectors showed significantly decreased levels of intracellular cAMP after stimulation, in approximately half max value compared with cells expressing wild-type AVPR2 (Figure 4B). These results indicated the dysfunction of mutated AVPR2, despite normal localization and maturation processes.

### Follow Up

Based on the clinical information, and the results of genetic sequencing and functional analysis, the diagnosis of CNDI was established. To relieve the symptoms of polyuria and polydipsia, the proband was treated with hydrochlorothiazide 12.5 mg twice daily and potassium citrate oral solution (potassium citrate monohydrate 7.3 g/dl) 5 mL twice daily at the age of 3 years 3 months old (at his first follow-up) for 2 weeks, and then switched to one tablet of compound amiloride hydrochloride (containing amiloride 2.5 mg and hydrochlorothiazide 25 mg per tablet) per day. After the treatment, his daily liquid intake decreased to 3,000 mL, so as to his urine output.

Four months after treatment with compound amiloride hydrochloride, his symptoms of polyuria and polydipsia was improved remarkably, but his body height increased only 1.1 cm (3.3 cm/year). His IGF-1 level was still below 25 ng/mL (<-2.42 SDS). Therefore, we added recombinant human growth hormone (rhGH) to treatment regime to improve growth retardation since 3 years and 7 months old. At the beginning, his body height was 85.5 cm (−4.31SDS) and rhGH was started with 0.14 IU/kg every other day. After 5.5 months treatment, his height was 90 cm (−4.01 SDS) and his IGF-1 increased to 64.75 (−1.19 SDS). Then rhGH dose was titrated up to 0.12 IU/kg daily, his body height increased to 95cm (−3.15 SDS) after additional 6 months treatment. 8 months after about 0.13IU/kg daily growth hormone treatment, his body height was 101cm (−2.71 SDS). Totally, he had been on rhGH treatment for 18.5 months, and his body height increased by 15.2 cm after the whole treatment period (9.86 cm/year).

## Discussion

CNDI is a relatively rare inherited disease due to renal resistance to AVP. The chief symptoms of CNDI at time of admission were faltering growth, vomiting, polyuria/polydipsia and febrile illness with hypernatremic dehydration and the median age of diagnosis was 0.6 years (2, 18). The majority of CNDI cases are caused by *AVPR2* mutation, and the rest are caused by *AQP2* mutation.

In this study, we identified a novel variation in *AVPR2* gene in a four-generation Chinese family with NDI by whole-exome sequencing. Not only the proband (case IV-1), the proband's brother (case IV-2) and maternal cousin brother (case IV-3), but also the proband's maternal grandfather (case II-2) and the paternal cousin of the proband's mother (case III-6), had a long history of polyuria and polydipsia. All of the affected members were males with a missense mutation in exon2 (c.530T > A) of *AVPR2* gene, suggesting co-segregation of the variation with polyuria and polydipsia. The mutation has not been found in population databases and the effect of the missense mutation was predicted to be a disease-causing mutation by PROVEN, SIFT, PolyPhen-2 and Mutation Taster Mutation. Functional analysis also showed that the intracellular cAMP concentration was lower in mutation AVPR2 transfected cells than wild type cells stimulated by AVP. According to the criteria supplied by The American College of Medical Genetics and Genomics (ACMG) (19), this mutation could be classified as likely pathogenic (functional characterization-PS3, population data-PM2, family co-segregation-PP1, *in silico* predictions-PP3, and family phenotypes highly specific for gene-PP4).

AVPR2 belongs to a class of G protein-coupled receptors (GPCRs), composed of 371 amino acids with seven transmembrane helices, three extracellular and three cytoplasmic loops (20). Its complex three-dimensional structure is generated in the endoplasmic reticulum (ER) and Golgi apparatus by posttranslational modification, and mutated protein with a wrong structure would be retained in ER (21, 22). AVPR2 is expressed in the cell surface and couples to AVP. It is essential for the kidney's ability to concentrate the urine and maintain water balance in the kidney. Mutations have now been identified in each domain of AVPR2. However, compared with the extracellular or intracellular domains, on a per nucleotide basis, about twice as many mutations have been reported in transmembrane domains (7). In our pedigree, the missense mutation I177N was located in transmembrane helix 4 (TM4) close to the cytoplasm and was conservative in mammalian orthologs. Several conservative or not conservative residue mutations TM4 of AVPR2, including those at positions A163, W164, A165, S167, L169, L170 and P173, have been identified and are supposed to cause CNDI (HGMD Professional 2021). These results indicated the importance of TM4 for AVPR2 function (Figures 2A,B).

Defects in AVPR2 have been classified into three types:(1) type I mutant receptors (protein expression disorder), which result in decrease, truncated, or even null protein expression due to defects in transcription, mRNA processing, and translation; (2) type II mutant receptors (localization disorder) with defective intracellular transport that cannot reach the cell surface and are trapped in the interior of the cell; (3) type III mutant receptors (functional disorder), which reach the cell surface but display ligand-binding disorder, G-protein coupling disorder or constitutive endocytosis and are unable to induce normal cAMP production (6, 10, 21). Among these types, type II mutant receptors are the most common and occur in ~70% of NDI cases (10). In our cases, the mutation of I177N in AVPR2 resulted in change of the hydropathic character of AVPR2 protein predicted by the Kyte-Dolittle Hydropathy Plot program (Figure 2C), therefore may cause the disorder in the ligand-binding or G-protein coupling ability of the receptor. The expression analysis revealed similar trafficking and plasma membrane localization of AVPR2-I177N with wild-type AVPR2 (Figure 3). Moreover, the functional study showed a blunted cAMP response to AVP in the mutant AVPR2 compared with the WT receptor (Figure 4B). Thus, our results suggested that the identified mutant was dysfunctional with normal cellular expression and distribution, and could be classified as a type III mutant receptor.

**Figure 5:**
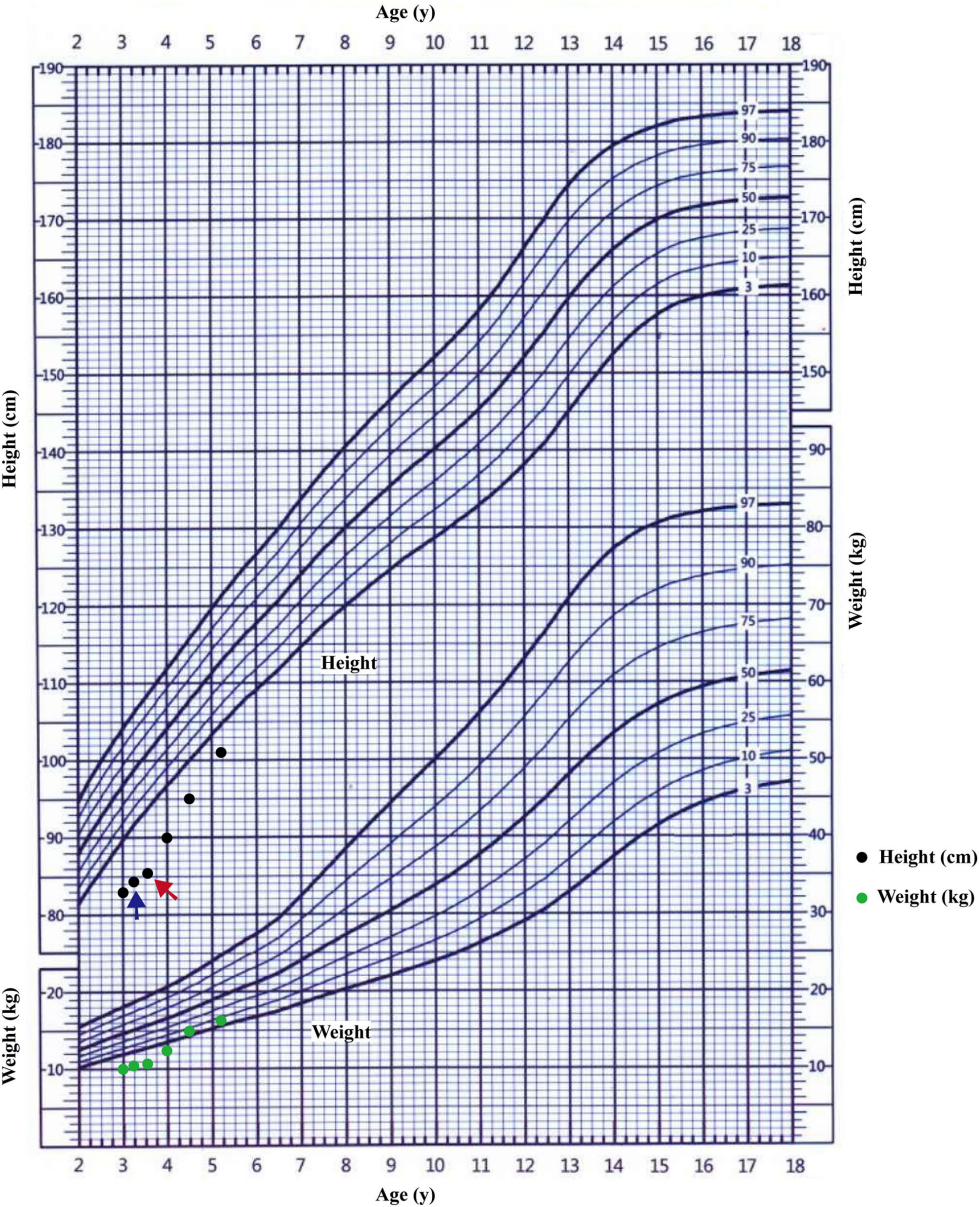
The growth curve of the proband. The starting treatment with thiazide diuretics and potassium supplementation was showed by blue arrow; the starting treatment with rhGH was showed by red arrow.

Most pathogenic variation of *AVPR2* related with CNDI are missense mutations that disrupt receptor function at various levels (23), therefore clinical symptoms and the response to AVP can be diverse. Faltering growth is one of the most common symptoms of CNDI at time of admission (2, 18). Available data showed that bone ages of CNDI were generally in accordance with chronological ages (24). Slowed weight gain and linear growth may result from a child's strong preference for water, limiting the intake of more caloric liquids or solids (6). The pediatric CNDI patients with confirmed *AVPR2* variations had a significantly low height SDS, but early diagnosis and rigorous treatment with a low-salt diet, prostaglandin synthesis inhibitors and thiazide diuretics showed normal adult heights (2, 25). This scenario was in agreement with our patients that the body heights of the proband's maternal grandfather and maternal uncle were normal. However, there was still a group of individuals from a large NDI cohort did have a low final adult height (SDS < −2.0), which was not in the normal range (25). There were few reports studied the endocrine hormones in CNDI patients. One study investigated the treatment of rhGH in a CNDI case cause by *AQP2* variation, but the endocrine hormones and the response of rhGH were not well-documented (26). In present study, the proband's height SDS before rhGH treatment was −4.31 SDS, and he had a low peak GH level as well as low IGF-1 level, suggesting a possible diagnosis of GH deficiency. As shown in the growth chart of the proband (Figure 5), after 4 months of the treatment with compound amiloride hydrochloride, his symptoms of polyuria and polydipsia was improved substantially, but his body height increased only 1.1 cm (3.3 cm/year). Given that the proband serum peak growth hormone (GH) concentration was low and his IGF-1 SDS < −2.45, we added rhGH to the treatment regime. With rhGH treatment, his IGF-1 level increased to 64.73 ng/mL (−1.19 SDS) and his growth velocity increased to 9.86 cm/year. These data strongly suggested rhGH treatment significantly improved the growth of the patients, but not compound amiloride hydrochloride. Our data implied that for the children with CNDI in combination of extreme short stature, it could be worthwhile to investigate the function of growth hormone axis, and rhGH treatment could be helpful to improve the final adult heights.

The current treatment approaches for CNDI focus on ameliorating the symptoms including management of dehydration and hypernatremia, prostaglandin synthesis inhibitors, thiazide diuretics, and a low-salt diet (16). However, CNDI is difficult to treat since these patients are resistant to AVP agents (at least partial), and there are no reasonable therapeutic options can be offered to patients with primary polydipsia (27). Several novel treatment approaches for patients with CNDI have been proposed, but little clinical data are available until now. These novel approaches focus on mutation-specific treatment such as the rescue of AVPR2 mutants by chemical chaperones or the bypass of defective AVPR2 signaling or the development of new molecules for binding with the mutated receptor to induce allosteric effect (1, 11). These potential therapeutic strategies targeting specific defects are largely rely on the understanding of the effects caused by mutated receptors. Therefore, it is important to elucidate the correlation between genotypes and phenotypes, and to investigate the function and molecular characteristics of the mutated receptors.

Overall, this study reports a novel mutation identified in a four-generation Chinese family with CNDI that caused by I177N mutation in AVPR2. We analyzed the pathogenic consequences of the mutation by using bioinformatic approaches and *in vitro* cellular experiments. The results suggested that the identified variation could be classified as a type III mutant receptor which reach the cell surface but are unable to induce normal cAMP production. So novel approaches for the variation should improve cAMP production, but not to change AVPR2 location. Additionally, for the pediatric CNDI patients with extreme short stature, investigations of the function of growth hormone axis could be important, and rhGH treatment could improve the final adult heights in these patients. This research provides more information for evaluating the effects of different mutations on AVPR2 function that may give some insight into designing effective treatment strategies for a given mutation.

## Data Availability Statement

The data presented in the study are deposited in the NCBI SRA data repository, accession number PRJNA769854.

## Ethics Statement

The studies involving human participants were reviewed and approved by Department of Pediatrics, Tongji Hospital, Tongji Medical College, Huazhong University of Science and Technology, Wuhan, Hubei, China. Written informed consent to participate in this study was provided by the participants' legal guardian/next of kin.

## Author Contributions

CZ and XL identified the clinical case. CZ and LH participated in the patient's clinical care and collected the data. SG and XL conceived and designed the experiments and contributed to the data analysis and interpretation, the drafting of the manuscript. DZ and SW performed bioinformatic analysis. SG and ZL performed cellular experiments. CZ and XL secured funding for this project, and approved the submitted paper. All authors contributed to the article and approved the submitted version.

## Funding

This study was supported by the National Natural Science Foundation of China (No. 81471519) and National Key R&D Program of China (No. 2018YFC1002404).

## Conflict of Interest

The authors declare that the research was conducted in the absence of any commercial or financial relationships that could be construed as a potential conflict of interest.

## Publisher's Note

All claims expressed in this article are solely those of the authors and do not necessarily represent those of their affiliated organizations, or those of the publisher, the editors and the reviewers. Any product that may be evaluated in this article, or claim that may be made by its manufacturer, is not guaranteed or endorsed by the publisher.
